# A dual design thinking – universal design approach to catalyze neurodiversity advocacy through collaboration among high-schoolers

**DOI:** 10.3389/fpsyt.2023.1250895

**Published:** 2024-01-10

**Authors:** Rachel K. Schuck, Lawrence K. Fung

**Affiliations:** ^1^Department of Psychiatry and Behavioral Sciences, Stanford University, Stanford, CA, United States; ^2^Department of Education, University of California, Santa Barbara, Santa Barbara, CA, United States

**Keywords:** neurodiversity, design thinking, universal design for learning, autism, ADHD, dyslexia

## Abstract

**Introduction:**

Neurodiversity describes the fact that humans all have different brains with unique qualities that contribute to society. Though understanding of neurodiversity is gaining traction among the general public, there remains considerable stigma and prejudice toward neurodiverse people. One way to combat these issues is to teach individuals about neurodiversity and encourage them to develop advocacy skills. Development of such knowledge is especially important for adolescents, as they have the capacity to make small (e.g., interpersonal interactions) and large (e.g., school-wide) impacts.

**Methods:**

Eighty-nine high schoolers participated in a two-week virtual summer camp in 2022; research consent/assent was obtained from 19 (11 neurodiverse/neurodivergent). Campers learned about neurodiversity, Universal Design for Learning (UDL), and Design Thinking (DT) through lectures from researchers and neurodivergent people, as well as group activities and discussions. Campers worked in small groups to design a neurodiversity advocacy project based on the principles of UDL and DT. Each group was facilitated by camp counselors–some of whom were neurodiverse–who were all committed to neurodiversity advocacy. Participants completed questionnaires about autism, ADHD, and dyslexia pre- and post-camp. Some also completed optional post-camp interviews.

**Results:**

Pre-camp stigma toward neurodiverse conditions was generally low. However, autism stigma was significantly higher than dyslexia stigma (*Z* = −2.24*, p* = 0.025). After camp, autism stigma decreased (*Z* = −2.98, *p* = 0.003;) and autism [*t*(13) = 3.17, *p* = 0.007] and ADHD [*t*(13) = 2.87, *p* = 0.013] knowledge improved. There were no significant changes in ADHD or dyslexia stigma or dyslexia knowledge. Participants reported enjoying collaborating with other campers and learning about UDL and DT. Thematic analysis of interviews generated four themes: Increased Understanding of Neurodiversity; Increasing Empathy and Becoming Less Judgmental; Creating a Neurodiverse Community; and More Awareness is Needed.

**Discussion:**

This pilot investigation suggests that a virtual summer camp can be effective in improving attitudes toward and knowledge of neurodiversity. Qualitative analysis indicated participants became more accepting after the camp, both in terms of being less judgmental toward neurodiverse people and more self-accepting among neurodivergent campers. Future research should investigate the long-term effects of such a program, particularly with diverse samples of students.

## Introduction

### What is neurodiversity?

Neurodiversity, at its most literal level, refers to the diversity of human neurobiology. Judy Singer, an Australian sociologist who coined the term *neurodiversity* in her 1998 thesis, likened neurodiversity to ecological diversity, highlighting that both conferred benefits to the human species and society (1). Apart from being a fact of biology, “neurodiversity” is often used when evoking the *neurodiversity paradigm* [or *approach* [es] (2)], which is a way of viewing the world in which there are no “right” kinds of brains, and people with different kinds of brains should be accepted and valued (3). Generally, people whose brains function within the norm and whose behavioral manifestations align with society’s social expectations are referred to as neurotypical. In contrast, people whose brain functions and behavioral manifestations deviate from the societal norm are referred to as neurodiverse or neurodivergent. (Note that we will use “neurodiverse” and “neurodivergent” interchangeably throughout this paper to reflect the varying preferences of individuals who fall under the neurodiverse umbrella. Similarly, we refer to both “neurodiverse conditions” and “neurodivergences”). Examples of neurodiverse conditions include autism, attention-deficit/hyperactivity disorder (ADHD), dyslexia, and Tourette’s syndrome.

The neurodiversity approach is in stark contrast to the medical model of disease/disability, wherein disability is located within an individual, who then requires treatment to ameliorate the disability or symptoms (4). For example, a child diagnosed with autism might be enrolled in therapy to get them to appear more like a typical child, for instance, by reducing their self-stimulatory movements and encouraging eye contact. While the neurodiversity approach is not synonymous with the social model of disability, it does have much more in common with it than the medical model. Under the social model, disability exists within society, and individuals with physical or psychological impairments are only disabled to the extent that society oppresses them (4). Thus, according to a strict interpretation of the social model, if society changes (for example, by creating curb cuts so wheelchair users can easily navigate sidewalks), the disability may no longer exist.

The neurodiversity paradigm aligns itself well with the social model of disability in multiple ways. For example, neurodiversity proponents highlight the ways in which societies are not created for neurodiverse people and thus contribute to their disablement (5, 6). They therefore stress the importance of environmental changes and accommodations in order to help neurodivergent people thrive. There are slight differences between the neurodiversity paradigm and the social model, however [though some do equate the two (6)]. In addition to being specifically focused on differing neurobiology (as opposed to any kind of disability, psychological or physical), there is room within the neurodiversity approach to support individuals above and beyond societal changes. This is especially true when those supports enhance quality of life (7). Indeed, some autistic people have echoed the common criticism of the strict social model that it can erase embodied feelings of disability (8, 9), for example, if someone has extreme sensory sensitivities. Similarly, Dwyer (2) argues that the neurodiversity approach shares similarities with the social-relational model of disabilities (10), where some individuals may benefit from both environmental accommodations and interventions targeted at the individual (though ultimately curing or normalizing should never be the goal). Some models of neurodiversity specifically center neurodivergent people’s strengths (11), such that interventions and supports are tailored to capitalize on what the individual is interested in and/or already good at [see also (12, 13)].

### Why is understanding neurodiversity important?

Neurodiverse people face tremendous stigma and prejudice in today’s society [e.g., (14–16)]. Such stigma cuts across all neurodivergences, though each neurotype experiences this differently. For example, adults with ADHD report high levels of public stigma and expected discrimination (14). Autistic adults report having to navigate stigma and the stereotype that autism is “bad” (15). Teachers and parents are more likely to perceive disability and have lower educational expectations if a student is *labeled* as having a learning difference compared to matched students who are not labeled as such (16).

This stigma and prejudice lead to ableism, wherein those seen as less “able” than others are discriminated against. Because the neurodiversity approach is based on the acceptance of brain differences, it has the potential to address the ableism that has been perpetuated by the application of the medical model to neurodiverse conditions. According to Link and Phelan (17), stigma occurs when labels are applied to differences among people, and those differences are associated with negative stereotypes. This then allows the creation of an “us versus them” mentality, which ultimately can result in negative effects due to discrimination. Chapman and Carel (18) argue that stigma toward neurodivergent people has led to society discrediting, disenfranchising, and excluding them from what society considers a “good life.”

Neurodiverse conditions are highly stigmatized, but many studies suggest that it is not neurodivergence (e.g., autism) itself that can lead to lower quality of life or well-being, but instead, lack of social support and/or acceptance (19–22). Nonetheless, many neurodiverse people are at risk of negative outcomes, likely due to these societal pressures and poor fit between individuals and their environments (2). For example, while some autistic people may prefer to interact with other autistic people (23), interactions with neurotypical people are inevitable at places like school and work. Thus, while young autistic people do report wanting and having friends, they also report difficulty trying to navigate neurotypical social norms, which can lead to feeling the need to change themselves in order to “pass” at being neurotypical (24, 25). Other research has found that young autistic people report experiencing neglect, rejection, and scorn at school (26), and are at high risk for bullying victimization (27). Adolescents with ADHD report being bullied, feel that society lacks empathy toward them (28), and are likely to experience peer rejection (29). Similarly, those with learning differences are more likely to struggle with interpersonal difficulties and report higher levels of loneliness and stress (see Al-Yagon and Margalit (30) for a review). Neurodivergent individuals may also be subject to self- or internalized stigma, wherein the stigmatized person accepts society’s view of them and sometimes perpetuates the stigma toward themselves and others (31). One way of combating such internalized stigma is via self-acceptance, which has been linked with better mental health (19) and increased self-efficacy and self-regulated learning (32) in neurodivergent samples. Another way of relieving such stigma is self-compassion, which was found to be associated with higher psychological well-being and lower depression symptoms in both autistic and non-autistic adults (33). Thus, the adoption of the neurodiversity approach – which emphasizes acceptance – may hold promise for reducing stigma among neurotypical people, as well as reducing self-stigma among neurodivergent individuals.

### Approaches to reducing stigma and prejudice

There are several approaches to reducing stigma and prejudice toward neurodivergent people. The ones highlighted below are awareness/acceptance programs, direct contact with neurodiverse people via inclusive settings, and programs specifically tailored to educate people about neurodiversity.

It should be noted that these approaches are in contrast to approaches that are solely focused on the autistic individual. For example, many intervention programs, including social skills programs, focus on teaching neurodiverse people skills that neurotypical people frequently use. For example, a host of social skills programs targeting autistic youth aim to increase their verbal and nonverbal initiations and responses to and engagement and sustained interaction with neurotypical peers [see Sutton et al. (34) and Whalon et al. (35) for two reviews of social skills interventions]. These are undoubtedly beneficial skills to learn, and reviews/meta-analyzes have found evidence of the benefits of these interventions (34–36). However, targeting only the neurodivergent child is an issue for multiple reasons. First, this implies that there is something inherently wrong with neurodivergence, which contrasts the tenets of the neurodiversity approach (2, 3). Second, teaching autistic individuals to act neurotypical in order to fit in can lead them to “mask” or “camouflage” their true selves (37, 38), which has been found to correlate with multiple negative mental health outcomes in autistic people (39, 40). Therefore, even if children could learn to perfectly enact every social skill in order to blend in with neurotypicals, this would likely have a negative effect on their quality of life. It should be noted, however, that “unmasking” can be viewed as a privilege not extended to non-White autistic people, who may feel that masking helps keep them safe, for example, with regards to Black autistic individuals who mask while interacting with the police (41). Lastly, focusing solely on the neurodivergent person ignores the fact that social interaction is a two-way street–why should, for example, children on the autism spectrum have to learn so much about neurotypical social interactions while neurotypical students are rarely expected to learn about the ways autistic people prefer to interact? This is an example of the double empathy problem (42), wherein a lack of understanding between different groups leads to mismatched expectations and difficulty in interacting. With regard to autistic people, they are expected to have enormous empathy for neurotypicals and accommodate their needs, whereas the reverse is rarely evident. As such, autistic people are often taught neurotypical social skills, but neurotypical people are rarely (if ever) taught about how autistic people prefer to interact socially. It is for these reasons that it is imperative that neurotypical people learn more about neurodivergent people.

### Awareness/acceptance programs

While research suggests many youth and young adults have a basic awareness of autism (43, 44), there are still many reported inaccuracies [(45, 46); see (47) for a review]. Even in studies that have found high awareness and understanding of autism (48), such awareness is not necessarily from a neurodiversity perspective (e.g., there is little emphasis on acceptance of differing brains, masking, etc.). Similarly, there exist misconceptions and gaps in knowledge regarding ADHD (49), for example that ADHD is caused by sugar intake or failure to recognize the genetic heredity of ADHD.

Three reviews of autism acceptance/awareness/anti-stigma interventions for non-autistic peers were recently conducted (50–52). Programs reviewed in these studies varied widely, though all shared the goal of increasing understanding of autism. Some were short, one-off interventions [e.g., showing a video about an autistic child (53) or a self-paced online training (43)]. Other programs were longer, including those implemented in classrooms over a period of weeks (54). Overall, while many interventions reviewed show promise, especially with regards to self-reported knowledge about autism and, to some extent, attitudes toward autistic individuals, peers’ behavioral intentions seemed less easily modulated across studies. It is also important to note that even when interventions purport to have the same goal (improving attitudes toward autistic peers), and even if they use the same outcome measures [e.g., the Adjective Checklist (55)], the effects of the intervention could vary drastically depending on the *framing* of the intervention. For example, Birnschein and colleagues (50) included peer-mediated interventions in their review. These kinds of interventions frame the neurotypical student as a helper, placing the neurotypical student in a position of power compared to the autistic peer. Similarly, Campbell and Barger (47) suggest that peer education and awareness programs be combined with peer tutoring or peer mentoring. Again, this frames autistic students as lacking and in need of help, as opposed to focusing on building reciprocal relationships between equals who have different interaction styles. Indeed, Morris et al. (52) point out that some awareness interventions may actually be inadvertently stigmatizing, and Cremin et al. (51) highlight that few such programs have assessed social validity.

### Direct contact with neurodiverse people

Allport’s (56) contact theory posits that contact between groups (i.e., a majority “in-group” and a minority “out-group”) can reduce prejudice. Pettigrew and Tropp’s (57) meta-analysis of studies based on contact theory found that direct contact with the out-group can reduce stigma and prejudice via increased empathy and decreased anxiety toward, and – to a lesser extent – knowledge of the out-group. When applied to neurodiversity, this would suggest that interacting with neurodivergent people can improve neurotypical people’s attitudes toward them. Indeed, Rademaker et al.’s (58) review found that both contact with and information about peers with disabilities contributed to improved attitudes among non-disabled children in inclusive education settings. Another recent meta-analysis (59) also found that inclusive education led to improved social effects, such as greater peer acceptance and less prejudice. Studies that have specifically manipulated direct contact (e.g., by implementing a buddy system) have also shown promise in improving attitudes (60). However, inclusive settings nonetheless run the same risks as some of the aforementioned awareness/acceptance programs; that is, even when they recognize that inclusion leads to the possibility of being friends with disabled students, peers without disabilities often see themselves as helpers or facilitators (61). This could explain why there is some variation in the findings in the above meta-analyzes [e.g., (58–60)]. Social inclusion and contact by themselves are thus not enough to ensure positive attitudes develop [and could in some circumstances lead to an “us versus them” mentality (62)].

### Interventions designed to promote neurodiversity

It has been argued that adopting the neurodiversity approach can have beneficial effects across a variety of contexts, including school (12), early intervention (63, 64), and even with regard to reducing stigma toward people with addiction issues (65). These arguments are bolstered by Kim and Gillespie-Lynch’s (66) finding that those with less knowledge of autism and less endorsement of the neurodiversity movement report higher stigma toward autism. This stigma is not inconsequential—in Cage and Troxell-Whitman’s (40) online survey study, feeling accepted by others was significantly related to reduction in depression and stress in autistic adults. Personal acceptance was also a predictor of depression and stress. Similarly, college students with learning disabilities reported greater academic self-esteem and greater career aspirations if they saw themselves through a neurodiversity lens as opposed to the medical model (67). Therefore, a great understanding of and positive attitude toward neurodiversity is likely to lead to greater acceptance (both external and personal) and well-being via a reduction in stigma.

Gillespie-Lynch and colleagues (46) created an online intervention to increase knowledge about autism and improve attitudes toward autistic people among college students. However, the authors recognized two important aspects of many previous interventions: (a) behavioral intentions often did not change attitudes toward autistic people, suggesting that neurotypical people were no more likely to want to hang out with an autistic person than prior to the intervention; and (b) the language used to explain autism in other studies may not be very useful in decreasing stigma [e.g., an autistic person described as having “something wrong with his brain” (53)]. The training included details regarding diagnostic changes from the DSM IV to DSM 5, issues of diagnosis in females, cultural factors including stigma, heterogeneity of intelligence in autistic people, etiology, empathy, challenges facing adults on the spectrum, and neurodiversity. Though the training was developed by a non-autistic researcher, the research team included an autistic scholar and self-advocate who provided feedback on the training. Stigma [assessed using an adapted version of the Social Distance Scale (68)] significantly decreased from pre- to post-test. At the item-level, there was significantly more willingness to collaborate with and marry/date someone on the spectrum after the intervention (though the item assessing stigma regarding romantic relationships was rated highest of all at both timepoints). Autism knowledge also increased after the training (with effect sizes larger than for stigma), though participants’ open-ended definitions of autism did not improve. The same training was also successful at increasing knowledge and to some degree reducing stigma in a sample of Japanese college students (69).

While Gillespie-Lynch et al.’s (46) training utilized an inclusive, neurodiversity-affirming framework, the team still felt that the training was lacking autistic input. They therefore conducted a study looking at differences between a training that was developed with autistic individuals using a participatory framework and one that was developed primarily by non-autistic people (70). The non-participatory training was adapted from the initial 2015 training. The participatory training was developed with multiple autistic college students (including one non-speaking student) and included videos of the students throughout the training. While some of the information presented was similar to the non-participatory training, the participatory one especially emphasized topics that were of importance to the autistic students, such as gender and motherhood in autistic individuals. The two trainings were matched on length and number of videos (the non-participatory training included TEDTalks and other informational videos in place of the student videos). While both trainings resulted in increased knowledge, reduction in stigma, and improvements in attitudes toward inclusion, the impact of the participatory training was greater than that of the non-participatory one.

Several studies have documented the promise of teaching individuals about neurodiversity through the lens of Universal Design (UD) (71). UD is a way of designing products, spaces, and materials such that they are accessible to everyone from the get-go, as opposed to having to provide retrofits to ameliorate non-accessibility. When applied to learning, UD (or Universal Design for Learning; UDL) focuses on the fact that we all have different brains with different strengths (72), and no one-size-fits-all approach will work for all people, an approach that is very much in line with the neurodiversity perspective. In a study designed to improve the way university educators teach autistic students, Waisman and colleagues (73) developed an asynchronous, online training about autism and UD. After participants reviewed the two modules (one about autism, one about UD), their reported knowledge of autism improved, and stigma decreased. Most participants also felt that they understood more about neurodiversity after the training, and their definitions of autism were more in-line with the neurodiversity perspective.

Similarly, during the early days of the COVID-19 pandemic, Lambert and colleagues (74) taught math educators about UD in order to help them design educational materials that would be accessible while teaching online. Recognizing that the Universal Design for Learning framework lacks clear guidance on how to actually implement inclusive curriculum, Lambert and colleagues (74) also utilized the principles of Design Thinking (DT), which is a step-by-step, iterative approach to designing. The DT steps include empathize (what do my users actually need/want?), define (what exactly is the problem I am going to try to solve?), ideate (how might I solve this problem?), prototype (develop potential solutions), and test (how would this solution work for the users?). Participants not only learned about disability, UD, and DT, but they also designed hands-on projects in small groups. After the 6-week course, participants in Lambert et al.’s (74) study reported shifts away from deficit-conceptions of students with disabilities and recognized that a major key to working with disabled individuals was to listen to their needs (75).

Thus, didactic teaching about disability and neurodiversity, emphasizing intentional design, and giving opportunities to create tangible materials using the principles of UDL and DT appear to be effective ways of improving knowledge of and attitudes toward neurodiversity.

### Current study

There is research to support a variety of interventions to reduce stigma and prejudice and improve the quality of life among neurodivergent people. These include awareness/acceptance interventions, inclusive environments that encourage direct contact among people with and without disabilities, programs that include explicit teaching about neurodiversity, and trainings that emphasize hands-on advocacy projects via UDL and Design Thinking. However, no studies have combined all of these approaches in order to teach *in-vivo* about neurodiversity advocacy while also providing direct contact with neurodivergent people. Adolescents are in a unique position to enact both formal advocacy (e.g., through school clubs or volunteer opportunities) as well as have a sustained direct impact on neurodivergent peers through day-to-day interactions at school. Learning about neurodiversity may improve such day-to-day interactions, and an understanding of how to apply UDL via Design Thinking might help more formal advocacy efforts.

The current multimethod study aimed to preliminarily assess the effectiveness of a two-week summer camp designed to improve high schoolers’ attitudes toward and increase knowledge of neurodiversity. The camp consisted of both didactic sessions intended to teach participants about a variety of issues related to neurodiversity and a hands-on advocacy project. Similar to the Lambert et al. (74) study, the camp project incorporated principles of UDL and DT in order to guide participants in designing something that could benefit the neurodiverse community. The following research questions guided the study and its analysis:

- What impact does the summer camp have on participants’ self-reported stigma toward and knowledge of neurodiverse conditions, such as autism, ADHD, and dyslexia?
- What do participants feel are the best parts of the camp? What suggestions do they have for the future?
- In qualitative interviews, how do participants discuss their experiences in the camp? What take-aways are there?

## Method

### Participants

Participants were recruited from the pool of high school students who had already signed up to participate in the Stanford Neurodiversity Project - Research, Education, and Advocacy Camp for High Schoolers (SNP REACH) in Summer 2022. Of the 89 campers (about one third neurodiverse), parental consent was obtained for 23 campers. Of those 23 campers, 19 (12 female, 5 male, 2 non-binary) agreed to participate (18 provided written assent; 1 participant who turned 18 years old after their parent consented provided written consent). Eight participants (42.1%) identified as Asian, five (26.3%) as White, three (15.8%) as Mixed Race, one (5.3%) as Hispanic, one (5.3%) as Middle Eastern, and one participant did not fill out the question about race/ethnicity. For both race/ethnicity and gender, participants were presented with multiple-choice options and asked to check off all that identified with; if “other” was chosen, participants could write-in how they identified. Participants ranged in age from 14 to 18. Eleven (57.9%) identified as neurodiverse/neurodivergent, with participants identifying as autistic (*n* = 6), as dyslexic (*n* = 3), having ADHD (*n* = 6), having dyscalculia (*n* = 1), and having dysgraphia (*n* = 1). Some participants had multiple diagnoses/identities. Three participants (including two who identified as neurodiverse and one who did not) indicated they had psychiatric conditions (depression and/or anxiety). Most (*n* = 14) indicated they had neurodiverse friends or family members; three indicated they did not, whereas two participants were not sure. See Table 1 for full demographic information. Because our research questions were focused on the overall effects of the camp (not differences between neurotypical and neurodivergent campers), and due to our small sample, the group of campers was considered one sample.

**Table 1:** Participant demographic information.

	Neurodiverse campers (*n* = 11)	Neurotypical campers (*n* = 8)	All campers (*n* = 19)
Gender			
Female	5	7	12
Male	5	0	5
Non-binary	1	1	2
Diagnosis^a^			
Autism	6	0	6
Dyslexia	3	0	3
ADHD	6	0	6
Dyscalculia	1	0	1
Dysgraphia	1	0	1
Anxiety	2	1	3
Depression	0	1	1
OCD	1	0	1
Neurodiverse			
Personal Contact			
Yes	9	5	14
No	1	2	3
Unsure	1	1	2
Average Age	16.11	15.88	16.00

^a^Self-reported diagnosis (participants could choose from a list of potential developmental disability and mental health diagnoses or write-in something else).

All 19 participants completed the baseline survey. Fourteen completed post-camp surveys (9 neurodiverse), and nine (4 neurodiverse) completed at least one interview. Most of the participants who completed an interview also completed the post-camp surveys, though two of the neurotypical participants who completed interviews did not, as they preferred to talk via Zoom than fill out online surveys.

### Procedure

Approximately 2–4 weeks before the start of the camp, an email was sent to all campers and their parents inviting them to participate. The email included a link to an online consent form for parents to fill out. Once parents consented, parents were sent a link to the child assent form and asked to share it with their child. Once assented, participants were asked to fill out a series of questionnaires before the camp. All 19 participants filled out baseline questionnaires (though one did so during week 2 of the camp). During the two-week camp, participants did not complete any research activities. Immediately after the camp, participants who completed baseline questionnaires were asked to complete post-camp questionnaires; 14 campers completed post-camp questionnaires. They were again asked 3 months later (10 completed follow-up questionnaires). Participants were also asked to participate in optional Zoom interviews at both follow-up timepoints. Nine campers (4 ND) agreed to the interview at one or both timepoints.

### Intervention: SNP REACH

The SNP REACH is a summer camp for high schoolers to learn about neurodiversity and collaborate on neurodiversity advocacy projects. The camp was started in 2019. SNP REACH lasted 6 hours per day, Monday through Friday, for 2 weeks. In 2022, SNP REACH was conducted entirely online via Zoom and Canvas. There were two cohorts of participants, one with 42 campers and the other with 47 campers. In each cohort, campers were split into small groups of 7–8. Each group was assigned 1–2 counselors to help facilitate discussion and guide their projects. Counselors included high school students who attended SNP REACH in previous years, undergraduate and graduate students, and recent college graduates. Most groups had two counselors. Younger, less experienced counselors (e.g., high school students and undergraduates) were paired with those who were older and more experienced counselors so that each group was led by counselors with a wide range of expertise. In the groups where there was only one counselor, the counselor was more mature and experienced (e.g., a graduate student). Five of the twelve counselors identified as neurodiverse/neurodivergent. Because there were fewer neurodivergent than neurotypical camp counselors, most groups had two neurotypical counselors, whereas some had mixed-neurotype counselors. All were involved in neurodiversity advocacy in some capacity. All camp counselors received an 8-week training (1 h, once a week) on neurodiversity, UDL, and DT before the camp started. Camp counselor trainings were led by the senior author.

**Figure 1:**
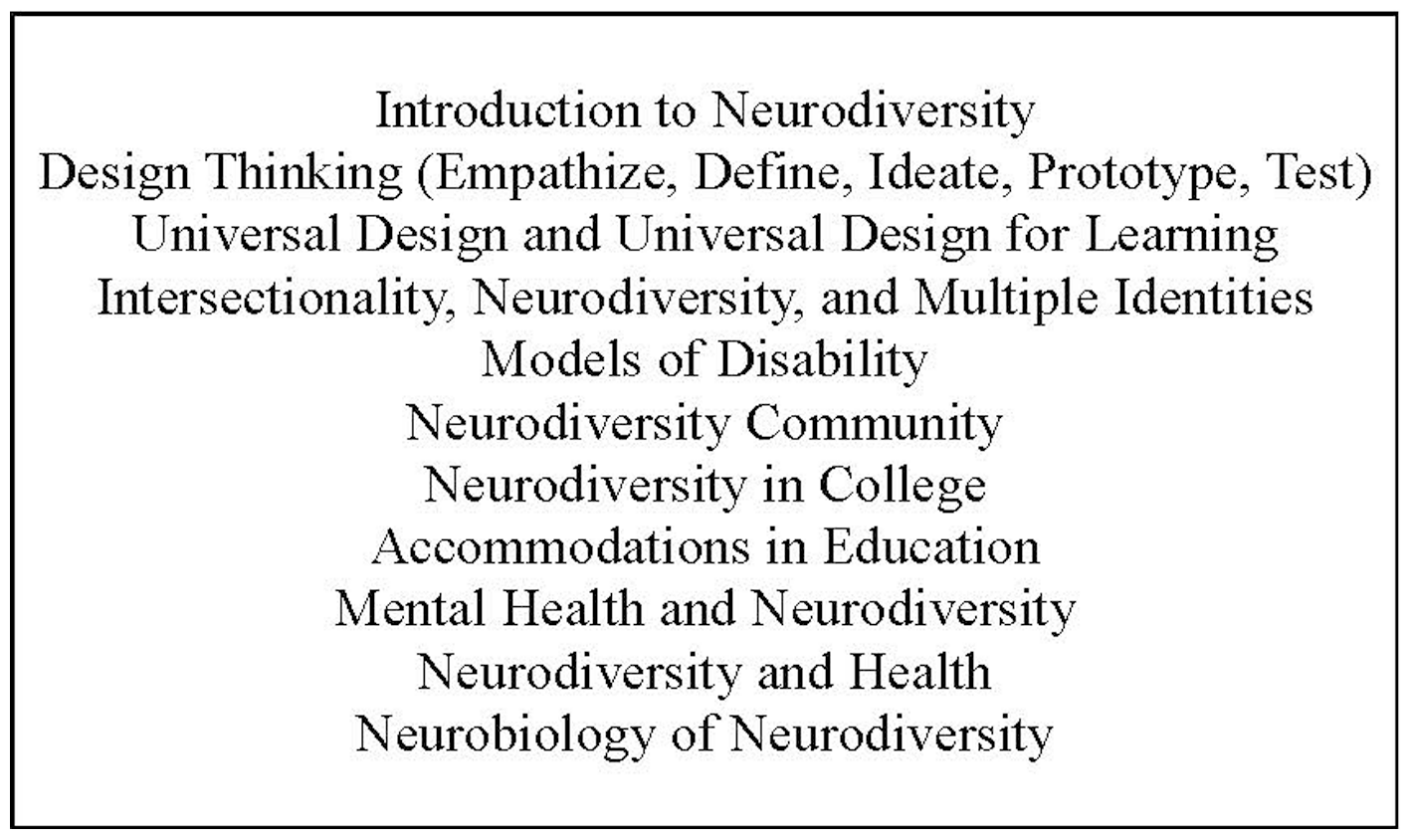
Topics covered at SNP REACH.

Day-to-day camp activities included large group activities such as expert lectures and workshops led by camp counselors as well as small group discussions and project work time (see Table 2 for an example camp day and Figure 1 for an overview of camp topics).

**Table 2:** Example SNP REACH Daily Schedule.

Time	Activity	Delivered by	Format
9:00–9:10 AM	Daily check-in	Camp director	Large group
9:10–10:30 AM	Lecture: neurodiversity in college	Content Expert (e.g., adjunct professor and social worker)	Large group
10:30–10:40 AM	Break		
10:40–11:40 AM	Discussion/activity: accommodations in education	Camp counselor	Large group and small group break-outs
11:40 AM–12:25 PM	Lunch break		
12:25–1:25 PM	Project-based learning: design thinking: prototype	Camp counselor	Large group and small group break-outs
1:25–1:35 PM	Break		
1:35–2:35 PM	Student group work time	Facilitated by camp counselors	Small groups
2:35–3:00 PM	Wrap-up	Camp director	Large group

### Expert lectures

One large group lecture was given via PowerPoint presentation daily at the beginning of camp. The initial lecture was given by Dr. Fung and focused on introducing campers to neurodiversity. Guest speakers gave the remaining lectures on topics such as inclusive playground design, disability law, lived experience of neurodiversity, neurodiversity advocacy, disability support for college students, neurobiology, and mental health. Experts included researchers and clinicians as well as neurodivergent students and advocates.

### Workshops

Workshops covered topics related to the morning’s lecture, including universal design for learning and accommodations, models of disability, health, and positionality. Additional daily workshops on the principles of design thinking were conducted in the afternoon. Most workshops were run by camp counselors. Workshops usually started by the camp counselor giving a short presentation to the whole group on an extension of the morning’s lecture (e.g., a workshop on accommodations followed the morning’s lecture on neurodiversity in college). After the short presentation, breakout rooms were utilized so that each small group could complete brief activities and answer discussion questions (e.g., in the context of the accommodations workshop, review different university Disabled Students’ Program webpages, and discuss what kind of accommodations might work best for them). Workshops usually ended with each group sharing what they discussed with the entire camp.

### Group advocacy project

The culmination of the camp was each group’s neurodiversity advocacy project. The purpose of these projects was to give the campers the opportunity to apply what they were learning in a hands-on way that could be applied to real-world settings beyond the camp (e.g., at their school or in their own communities). Groups were instructed to follow the design thinking process to create a product to address an issue relevant to neurodiversity. Daily DT workshops throughout the camp guided campers through the design process. Additional time was given every afternoon for groups to work freely on their projects, facilitated by their counselors. After going through the first DT steps (empathize, define, ideate, prototype), groups were encouraged to test their prototypes with potential users. Many groups sent out their prototypes (including magazines, websites, cookbooks, etc.) to members of the Stanford Neurodiversity Project Special Interest Group to get feedback. All groups presented their projects to the whole group on the last day of the first week and the last day of the camp. Families were invited to attend final presentations on the last day of camp. Groups were encouraged (but not required) to continue their advocacy projects beyond the end of camp.

### Measures

This study used multiple methods to answer our research questions: questionnaires (both standardized, validated instruments as well as open-ended questions about the camp, which were designed by the researchers) and qualitative interviews. Questionnaires were the same at all time points, with the exception of the open-ended questions. Interviews were only conducted at post-camp and 3-month follow-up.

### Open-ended questions

Participants were asked open-ended questions such as: What was your favorite part of the camp? What was the most important takeaway from camp? Do you have any suggestions for how to improve the camp in the future?

### Social distance scales

Three SDS’s were included: one about autism, one about ADHD, and one about dyslexia. Each SDS contained 10 questions about whether one would be willing to participate in different activities (e.g., *I would be willing to have lunch with an autistic person*). The SDS was originally developed by Bogardus (68), though the current version was adapted by Gillespie-Lynch et al. (70) to focus specifically on autism. Half of the items were reverse-scored. SDS item scores ranged from −2 to 2, with higher scores indicating more stigma. The autism-focused SDS exhibited strong internal consistency across Gillespie-Lynch et al.’s three samples (α range = 0.85–0.90) (70); internal consistency was slightly lower in the current study (*α* =0.77). For the purpose of the current study, all items from Gillespie-Lynch et al.’s study were modified to also refer to individuals with ADHD and dyslexia. These SDSs had excellent internal consistency (ADHD: *α* = 0.94; dyslexia: *α* = 0.91).

### Participatory autism knowledge-measure

The PAK-M was developed by Gillespie-Lynch et al. (70) and taps into not just common knowledge about autism (e.g., that vaccines do not cause autism) but also topics that were deemed important to autistic collaborators (for example, masking: “*Autistic people who hide their autism symptoms are more likely to experience mental health challenges than those who are comfortable with their autism*”). PAK-M item scores range from −2 to 2, with higher scores indicating more knowledge. Nine items were reverse-scored. The PAK-M exhibited satisfactory internal consistency (α range = 0.74–0.86) across multiple samples in Gillespie-Lynch and colleagues’ study (70) and had similar internal consistency in the current study (*α* = 0.88).

### Scale of ADHD-specific knowledge

The SASK (76) is a 20-item instrument designed to assess understanding of ADHD. Each item is presented as a statement (e.g., *ADHD is a neurobiological, developmental disorder*) and participants can indicate whether they think the statement is true, false, or do not know. Items were scored a 1 if answered correctly and 0 for incorrect or “do not know” answers. Two items were removed for this study (*A combination of stimulant medication and behavior management is an effective treatment for ADHD; Teachers are often the first to recognize ADHD type behaviors and refer children for assessment*), as it was decided that these items were irrelevant for our adolescent sample. Of the 18 retained items, four items were reverse-scored. The SASK had satisfactory internal consistency in Mulholland’s study (*α* = 0.88) (76); it was almost as high in the current study (*α* = 0.75).

### Dyslexia knowledge scale

The 10-item knowledge subscale of Gonzalez’s (77) dyslexia scale was used. Each item is presented as a statement (e.g., *Dyslexia is a learning disability that affects language processing.*) and participants indicate whether they think the statement is definitely true, probably true, probably false, or definitely false, with items scored on a scale of 1–4. Six items were reverse-scored. Gonzalez found the entire scale to have acceptable internal consistency (*α* = 0.70) (77). Internal consistency for the knowledge scale in the current study was much lower (*α* = 0.54).

Average scores for each instrument were obtained for each participant at pre-camp and post-camp.

### Interviews

The post-camp interview focused on take-aways from the camp, how they saw neurodiversity advocacy as part of their life moving forward, things campers liked about SNP REACH, and suggestions for the future. Three-month follow-up interviews touched upon any neurodiversity-related activities since the camp ended and further reflections on the camp’s impact. All interviews were conducted via Zoom by the first author.

### Data analysis

#### Analysis of quantitative data

Questionnaire data was first assessed for normality. The Shapiro–Wilk test indicated that all three SDS’s were non-normally distributed, whereas the knowledge scales were normally distributed. Thus, comparisons of baseline stigma and pre-post changes in stigma were assessed using Wilcoxon signed-rank tests, whereas pre-post comparisons of knowledge scales were assessed using paired samples t-tests. Due to the small sample and the exploratory nature of these analyzes, significance level was set at *α* = 0.05 and was not adjusted for multiple comparisons. Cohen’s *d* was used as a measure of effect size.

### Analysis of qualitative data

Two types of qualitative data were generated. First, written responses to open-ended questionnaire items (e.g., What was your favorite part of the camp? What was the biggest takeaway? What could be improved in the future?) were reviewed for common answer choices (e.g., group work, advocacy project, etc.). These answers were then quantified by tallying the number of participants who gave the most frequent answers.

Second, interviews were analyzed using reflexive thematic analysis (78, 79). According to Braun and Clarke, thematic analysis includes the following six steps: familiarization, generating codes, constructing themes, revising themes, defining/naming themes, and producing the report. All interviews were recorded via Zoom and the interviewer (the first author) took notes during each one. The automated transcripts generated by Zoom were then reviewed and edited by the first author while watching and listening to each video recording. After this initial familiarization with the data, the first author then read through all interview transcripts and created an analytic memo about each interview (80). These memos were then reviewed altogether, and an initial coding scheme was developed based upon commonalities throughout the interviews. After codes were generated, they were then applied while re-reading each interview transcript. Code names and meanings were continually updated during the coding process. Themes were then generated based on the codes. All themes were then reviewed by the senior author for peer debrief in order to assess credibility of the findings. The extensive data familiarization process and comprehensive audit trail enhance the trustworthiness of the analytic process. Additionally, both authors exercised reflexivity by recognizing their own values and positionalities (see below for more information).

### Author positionality

Both authors take a neurodiversity approach to working with neurodivergent individuals and conducting research. The first author, a recent doctoral graduate with a PhD in Education, identifies as female, White, and neurotypical. Her research interests center around the acceptability of interventions for autistic individuals and teaching the general public about neurodiversity. She has a background in delivering naturalistic developmental behavioral interventions with autistic children and their families. She also served as a camp counselor during the 2022 SNP REACH. The senior author is an academic psychiatrist specialized in autism and neurodiversity. He identifies as male, Asian, and neurodiverse. He is the father of a neurodiverse individual. He has 14 years of experience seeing patients on the autism spectrum. He is an active researcher, educator, and program developer in the fields of autism and neurodiversity. He is also the creator and director of SNP REACH.

## Results

### Quantitative data

At baseline, campers reported relatively low stigma toward the three neurodivergent diagnoses (on a scale of −2 to 2, where higher scores indicate greater stigma: autism: -1.67 (SD = 0.40), ADHD: -1.73 (SD = 0.52), dyslexia: -1.79 (SD = 0.40)). Though reported stigma was generally low, there was a significant difference between participants’ autism and dyslexia stigma scores (*Z* = −2.24*, p* = 0.025), with participants reporting greater autism stigma. Other baseline stigma comparisons were not significantly different.

After the camp, stigma scores decreased for all three diagnoses compared to baseline. However, the decrease was only statistically significant for autism (*Z* = −2.98, *p* = 0.003; see Table 3). Knowledge of autism and ADHD also significantly changed, with participants reporting more knowledge after the camp [autism: *t* (13) = 3.17, *p* = 0.007; ADHD: *t* (13) = 2.87, *p* = 0.013]. Knowledge of dyslexia did not significantly change. Effect sizes for the significant changes ranged from *d* = |0.77|—|0.85|, indicating relatively strong effects.

**Table 3:** Average questionnaire scores at baseline and post-camp.

	Pre-camp mean	Post-camp mean	Test statistic^a^	Effect size^b^
Autism SDS (Stigma)	−1.67	−1.84	−2.98*	−0.80
ADHD SDS (Stigma)	−1.71	−1.86	−1.22	−0.33
Dyslexia SDS (Stigma)	−1.80	−1.89	−1.84	−0.49
PAK-M (Autism Knowledge)	1.16	1.37	3.17*	0.85
SASK (ADHD Knowledge)	0.69	0.82	2.87*	0.77
Dyslexia Knowledge	3.17	3.13	−0.87	−0.23

^*^Significant at the *p* < 0.01 level. SDS = Social Distance Scale (measure of stigma). ^a^For SDS’s, test statistic is a Z-score; for knowledge scales, test statistic is a t-score. ^b^Effect size for SDS’s is calculated by dividing the test statistic by the square root of the number of participants (*n* = 14); for knowledge scales, effect size is Cohen’s d. All SDS’s and the PAK-M are rated on a scale from −2 to 2; the SASK is rated as a 0 or 1 and scores indicate average number of answers correctly answered; the dyslexia knowledge scale is scored from 1 to 4. Only data for the 14 participants who completed pre- and post-camp data is displayed here.

Comparisons in pre-camp to post-camp changes in quantitative measures between neurodiverse and neurotypical campers are not presented here due to the very small sample size in each group and the fact that this was not one of our research questions. However, these analyzes can be found in the Supplementary materials.

### Qualitative data

#### Questionnaires

When asked what their favorite part of the camp was, 6/14 of the participants who filled out the post-camp questionnaires indicated their favorite part was collaborating, interacting, and/or discussing with their small group. Five individuals mentioned the lectures/speakers, three mentioned the group project, and two mentioned the final advocacy project presentations. When asked what the most important take-away from camp was, 7/14 participants indicated it was learning about UDL and DT, three indicated learning more about self-advocacy, and two indicated the strengths-based model.

Suggestions for how to improve the camp in the future included increased opportunities to get to know fellow campers, providing more guidance regarding the advocacy projects, and having more interactive sessions (as opposed to lectures) in the morning. Some participants also mentioned how it might be interesting to have an even greater diversity of speakers, such as having a young neurodivergent person plus their parent, an elderly neurodivergent person, and international neurodiversity advocates.

#### Interviews

Thematic analysis of the post-camp and 3-month follow-up interviews generated four themes: Increased Understanding of Neurodiversity; Increasing Empathy & Becoming Less Judgmental; Creating a Neurodiverse Community; and More Awareness is Needed. Each theme is discussed in more detail below.

#### Increased understanding of neurodiversity

Five of the nine campers who completed an interview mentioned how the camp broadened their understanding of neurodiversity. This was an especially salient point, given that many of the campers were already familiar with neurodiversity before starting the camp. As one neurodivergent camper stated: I definitely feel like I learned a lot about a topic which I already kind of knew a large amount about…like the amount of difference there is between different types of neurodiversity – like one thing I really never actually heard of, which really surprised me that I had not heard of this, is nonverbal autism.

Another camper, who is neurotypical, mentioned how she realized how common different neurotypes were: “It definitely broadened in my view, beyond just ADHD and autism and to see how common it is and how many different ways it can manifest.” An autistic camper also mentioned that her view of neurodiversity was broadened due to the interactions with other neurodivergent campers: “I’m just maybe a little more broadened a little bit just because I met more people and people with diverse conditions like, I met people other people with autism. And then I met people with ADHD, people with dyslexia, you know, people who do not have any brain differences.”

Two campers mentioned how learning about neurodiversity from a factual, empirical standpoint was especially useful to their own conceptions of neurodiversity. For example, an autistic camper stated: “That’s why like [the] lectures about the studies are so important, because they are like empirical facts, instead of like people’s observations.” Another camper described how he felt validated in his own ideas after hearing about neurodiversity in such a way:

I feel like there were some ideas with support. I feel like I have a lot of like ideas, like – Oh, I have these ideas, they exist, but I do not have, like all this stuff to back them up. But I feel like now, I have a lot more to – like, I feel like I’ve definitely learned a lot, even if, like what I knew wasn’t changed, I guess.

Another two campers mentioned how their perspectives were broadened due to discussions around different models of disability. One described how they “learned a lot about the different models [of disability] and why each one is, has its strengths and weaknesses.” Another discussed how they thought “the main [takeaway] is just that things tend to be very oversimplified online and this camp really dove into like the depth of what we are talking about when it comes to, like the strength-based model.”

#### Increasing empathy and becoming less judgmental

Seven participants touched upon the idea becoming more understanding. This theme was broken up into two subthemes: Increasing Empathy and Becoming Less Judgmental. Three participants mentioned how the design thinking concept of empathy was important when thinking about how to best support neurodivergent people. A neurotypical participant explained, “I think that goes with any sort of community that’s misjudged, just breaking down those misconceptions. And that’s the biggest thing. Because if everyone can empathize with each other, right, so many of our problems would be gone.” Another participant, who was autistic, described how empathy could be used to design products that are more accessible and useful:

[The camp director] was wondering if we were actually addressing the pain points of neurodiverse students, so I learned that there’s basically, there’s no point of creating a product if it does not benefit the people you want to target. And you can think your product is all good and that it has the most advanced technology, but it does not end up benefiting the lives, or like addressing some of the suffering that people face on a daily basis, then there is no point of the product.

Similarly, another camper mentioned how they were rethinking the accessibility of some graphic design infographics they created over the pandemic:

We have to factor in things like font, we have to factor in things like audio versions and whatnot and I realized like, these online materials, I thought were more accessible, because of their format of them being online - I thought they are accessible but turns out maybe they are not. … so I think that’s my main takeaway, like needing to reach out to more people to actually be part of the design process.

Five participants (four of whom were neurotypical) described becoming more understanding in terms of being less judgmental after the camp. One camper described how she learned to be more supportive of people’s special interests: “I know it’s like impossible to completely have no prejudice but whenever I talk to people, making sure that I do not just, do not be rude or, let us see, like when people are really passionate about something, let them talk about it, be a good listener.” Another camper explained how the camp has allowed her to be more understanding of a friend with ADHD who was panicking about a class project and mentioned that her ADHD medications had worn off an hour before: “I feel like before the camp, I would have probably thought of this in a negative way, like, “Oh, my gosh! She takes meds!” -- but now it’s just like, I do not think much of it, and I think it’s completely natural.”

Two participants who tutor younger children commented on how the camp taught them to individualize their teaching such that each child’s strengths can be supported. Another participant described how she incorporated the strengths of people with ADHD into a class presentation about psychiatric diagnoses.

#### Creating a neurodiverse community

Three of the four neurodiverse campers discussed how they felt the camp taught them something about themselves as a neurodiverse person and/or created a sense of community among neurodivergent people. For example, a camper with ADHD discussed how her self-advocacy skills grew: “I always get worried about, like, if I’m telling people I need something, am I inflicting it on other people? So that was definitely one big barrier that I overcame…I have the confidence to go to the teacher and say, “Oh, I would like more time on this because my extended time was not met all the way.””

An autistic participant described how he felt that the camp allowed him to be “more accepting of some of the weird ways that I act.” He explained how seeing that there are other people in the world like him boosted his confidence and encouraged him to try to mask his autism less, which led to positive outcomes: “When I try to mask less, then I was less stressed, and I was actually more confident in talking to people, which is kind of counterintuitive. But, yeah I was more confident, more happy talking to people.”

Participants also felt that the camp provided a sense of community for neurodivergent people. An autistic participant stated that the camp is “kind of geared toward people like me so they have kinda like more similar traits to me than compared to other camps, so I guess there was a closer community here.” Another autistic camper explained how she felt included in the camp in a way that she does not always at school: “But it was definitely nice being in a group, where I was included and I could actually talk to any people in here…sometimes in groups in the past, like at school, it’s been harder to be included because I’m, you know, the quiet one and it’s difficult when you are with people who will not pull their weight, or who will not let you contribute. But our group was thankfully really nice about letting everyone contribute.”

#### More awareness is needed

Six participants brought up how there is little awareness or understanding of neurodiversity in the general population. A neurotypical participant explained that most teens.

“Have not been taught whatsoever about the community. And so our perceptions are filled up by whatever media we take in, or whatever biases or stigmas we hear around us…So anything, anything history-related, advocacy-related, just some sort of education about it – I think it would get rid of some of these biases and fill in with actual positive, factual information that I guarantee you very few high schoolers have right now.”

Another participant explained how his plans to incorporate a mentorship program into an existing neurodiversity club at his high school seemed unlikely given the club’s low membership: “And it could also be that no one really knows what neurodiversity is, which is probably what it is…I think most people kind of know that neurodiversity exists. I think it’s more like putting a name to that idea, and really like, I feel like just getting people more accustomed to the topic.”

Several other participants also discussed how schools were a great venue for teaching about neurodiversity. One participant had given presentations about autism during school assemblies and was planning more for the future, with the hope that they would inform both students and teachers. Another participant mentioned how incorporating the camp content into schools would help reach a wider audience and increase its impact: “I do not know if you guys have some sort of initiative to bring it to schools, but if you could, that’d be so cool because…if you could like, really magnify it, I think there’d be a lot of students that could benefit from it.”

## Discussion

According to pre-camp self-report, campers had relatively low stigma toward and high knowledge of three neurodiverse conditions that were seen as representative of the neurodiversity approach: autism, ADHD, and dyslexia. This is unsurprising, given that the campers self-selected into the camp based on an interest in the topic of neurodiversity. Nonetheless, there was a statistically significant decrease in autism stigma and increase in autism and ADHD knowledge from pre- to post-camp, though there were no significant changes in attitudes toward ADHD or knowledge of or attitudes toward dyslexia.

This finding could be explained by a variety of factors. First, there was more autism-specific (and to some extent ADHD) content in the camp, which is a likely byproduct of the neurodiversity movement being originally rooted in autism rights (81, 82). The significant decrease in autism stigma could also perhaps be attributed to the fact that stigma toward autism was higher than stigma toward dyslexia at pre-camp (autism and ADHD stigma was not significantly different, though it is possible that with more power from a larger sample, differences would have been detectable). This highlights that, even among teenagers who have an interest in neurodiversity, autism is perhaps especially misunderstood. This is in line with other research that autism is particularly stigmatized, even compared to other conditions (83, 84). Nevertheless, providing didactic instruction on neurodiversity and allowing participants to work together to create their own advocacy projects based on the principles of UDL and DT appeared to improve even the low level of stigma at pre-camp.

Additionally, the change in knowledge regarding autism could be attributed to the fact that Gillespie-Lynch et al.’s (70) autism knowledge measure was designed with an explicit neurodiversity perspective, whereas the other two instruments were not. This could thus provide evidence that the camp was successful in its goal of changing campers’ conceptions of disabilities from a neurodiversity—as opposed to medical model—point of view. Nonetheless, knowledge toward ADHD also significantly increased even though the instrument was not designed with neurodiversity in mind. While dyslexia knowledge did not improve, it is possible that this was due to the fact that the measure exhibited poor internal consistency in this study. This could be explained by the fact that the instrument was originally designed for teachers; high school students may not have enough background knowledge about dyslexia, which could have led to guessing on certain questions (e.g., most campers did not know that dyslexia is more common in boys than girls). This suggests that future iterations of the camp and similar programs should take care to include more information on neurodivergences that are less commonly discussed in the public sphere, such as dyslexia. Additionally, more research is needed into the construct of dyslexia knowledge among adolescents and how to accurately measure it.

One of participants’ favorite things about camp was interacting and working with others. Other future neurodiversity awareness/acceptance programs would thus likely benefit from interactive learning opportunities as opposed to only providing didactic instruction. Also, incorporating UDL and DT into the camp likely also impacted students’ feelings toward neurodiversity. In fact, increased empathy was one of the themes generated from participant interviews, with some participants even directly pointing out the importance of the empathize stage of DT. This is in line with research with special educators that indicated that empathy – i.e., listening to your user’s needs instead of assuming them – is a particularly compelling aspect of DT (74, 75). This perspective is distinctly different from other awareness campaigns that tell people how they should feel; instead, SNP REACH encourages campers to engage with and learn directly from neurodivergent people, keeping the focus directly on the user. Engaging with the principle of empathy thus likely allowed the campers to challenge their stereotypes and what they thought about neurodiversity. Additionally, many of the interactions between campers were likely cross-neurotype. In line with Contact Theory (52), prior research has shown that personal connections with disabled individuals is associated with more positive attitudes toward that disability (e.g. (85, 86)). It is thus likely that the camp’s success in reducing stigma was in part due to the fact that a substantial portion (about one third) of campers were neurodiverse. The immersive experience may have allowed participants to more fully assimilate the information they were learning didactically. Thus, future programs aiming to improve attitudes toward neurodiversity should aim to include participants of all neurotypes.

The qualitative findings from this study are particularly encouraging, as participants discussed positive changes in multiple areas. Not only did participants discuss feeling that they understood more about neurodiversity, but they also mentioned how they felt less judgmental after learning more about neurodiversity. Multiple participants described specific, real-world instances where they felt that they were more understanding of neurodiverse peers than they would have been prior to the camp. It is likely that these improved attitudes led participants to better interactions with the neurodiverse people they came into contact with [similar to (87), where non-autistic and autistic people endorsed an increased desire to hang out with one another after the non-autistic people participated in an autistic acceptance training compared to those who did not participate in the training]. Increases in empathy also likely improved actual behaviors, as evidenced by the participant who mentioned knowing she needed to redesign club advertisements to be more accessible. SNP REACH thus has the capacity to change actual behaviors, as opposed to only changing attitudes or knowledge. This is promising, as behavioral intentions (i.e., reported likelihood of engaging in positive behaviors toward neurodivergent people) have shown less malleability than attitudes or knowledge [see, for example, Cremin et al. (51)] for a review of autism awareness interventions).

The other highly impactful aspect of the camp, according to post-camp interviews, was the increased personal understanding of neurodiversity reported by three out of the four neurodiverse campers who completed an interview. Campers specifically discussed building a community with people like them, learning self-advocacy skills, and learning how to unmask and accept oneself. This is a particularly important finding, given that disability identity is related to self-esteem (88). Furthermore, masking has been linked with mental health issues in prior research (40, 89); if a program like SNP REACH is able to thus reduce masking and decrease social anxiety, there may be long-term benefits to mental health.

### Limitations

While the findings of this study are encouraging in terms of both reducing stigma toward and improving self-acceptance of neurodiversity, there are several limitations to consider. First, the study sample was fairly small, and our already small sample also experienced attrition from pre- to post-camp as the school year had started and campers were quite busy. It is thus difficult to extend our findings to other samples. We were also limited in the amount of subgroup analyzes we could do (e.g., differences between neurotypical and neurodivergent campers; differential effects of having a neurodivergent counselor). Additionally, it will likely be impossible for other people or organizations to exactly replicate our study, as the exact camp schedule with its specific speakers is unlikely to be duplicated. Also, while reflexive thematic analysis does not emphasize coding reliability (79), it is important to consider that other individuals with differing positionality may have extracted meaning from the qualitative interviews differently than the first author (who coded the interviews) and the second author (who reviewed the coding). Nonetheless, the mostly qualitative findings are not necessarily meant to be exactly replicated, and a camp of a similar nature could still be developed, particularly in other urban, diverse settings.

A perhaps more important limitation is the way our sample was recruited. All participants self-selected into the camp and had to partake in an application process. Therefore, all of our participants likely had knowledge of neurodiversity and at least some degree of interest in the topic. Thus, most of these highschoolers may be in less need of training on neurodiversity than those who did not sign up for the camp. It is therefore possible that the camp would not have had the same effect on students who were less familiar with or invested in the topic. Perhaps these students would have needed more background on neurodiversity and other models of disability, or perhaps an intervention that was spread over more than just 2 weeks. Therefore, as explicitly suggested by one of our participants, programs such as SNP REACH should be integrated into classrooms, where short- and long-term effects on all students (including those who are less motivated or knowledgeable) can be studied (see Alcorn et al. (90) for such a program that is being piloted in the United Kingdom). Similarly, all camp counselors were passionate about neurodiversity and highly motivated to support campers’ learning. Therefore, the transferability of our findings to other settings is potentially limited, as we speculate that SNP REACH will result in more optimal outcomes when instructors and camp leaders are carefully selected, and a significant proportion of campers are neurodiverse and interested in neurodiversity.

Finally, all of our data collection tools assessed explicit stigma and attitudes. Research has shown that implicit attitudes toward disability (91) are more difficult to change. Future research on programs such as SNP REACH should therefore include measures of implicit stigma, such as the implicit attitude test (92) in order to tailor such programs to target less overt demonstrations of prejudice. Future iterations of this research could also employ a waitlist-control group, such that campers’ pre- and post-camp changes can be compared to a control group who did not yet participate in the camp. It would also be helpful to understand how the effects of in-person SNP REACH might differ than virtual-delivery (as was the case in the current study).

## Conclusion

In conclusion, using UDL and DT as a framework for teaching adolescents about neurodiversity appeared to be effective at improving understanding and attitudes. While stigma toward and knowledge of autism shifted more on quantitative measures than dyslexia (and to some extent ADHD), qualitative findings suggest that participants felt the camp affected their perspectives toward neurodiversity in general, specifically with regard to being less judgmental and, for neurodivergent campers, being more self-accepting. Future research must assess the long-term outcomes of such programs and find novel ways of recruiting a wider variety of participants.

## Data availability statement

The raw data supporting the conclusions of this article will be made available by the authors, without undue reservation.

## Ethics statement

The studies involving humans were approved by the Stanford University Institutional Review Board. The studies were conducted in accordance with the local legislation and institutional requirements. Written informed consent for participation in this study was provided by the participants’ legal guardians/next of kin.

## Author contributions

RS and LF both designed the study. RS collected and analyzed the data and drafted the manuscript. LF supervised the study, provided feedback on analyzes, and edited the manuscript. All authors contributed to the article and approved the submitted version.
