# Supplementary material for: A dual design thinking – universal design approach to catalyze neurodiversity advocacy through collaboration among high-schoolers
Source: Front Psychiatry. 2024 Jan 10;14:1250895. doi: 10.3389/fpsyt.2023.1250895 (PMC10806093; doi:10.3389/fpsyt.2023.1250895)
Supplement: Supplementary file 1 [file Data_Sheet_1.docx]

**Supplemental Materials**

There were no significant differences in questionnaire scores between neurodivergent and neurotypical campers at pre-camp. When comparing pre-post difference scores, the only significant finding was that neurodivergent campers had a significant decrease in autism stigma from pre- to post-camp. None of the other pre-post differences were significantly different. These findings should be seen as highly exploratory, as the sample size is very small.

**Supplemental Table 1**

*Average Pre-Camp Questionnaire Scores: Comparison Between Neurodivergent & Neurotypical Campers*

|  | Neurodivergent Campers (n = 11) | | Neurotypical Campers (n = 8) | Difference Between ND & NT Campers |
| --- | --- | --- | --- | --- |
|  | Pre-Camp Mean | Pre-Camp Mean | | *t* |
| Autism SDS (Stigma) | -1.63 | -1.73 | | -0.52 |
| ADHD SDS  (Stigma) | -1.68 | -1.80 | | -0.48 |
| Dyslexia SDS  (Stigma) | -1.78 | -1.81 | | -0.16 |
| PAK-M (Autism Knowledge) | 1.10 | 1.08 | | -0.15 |
| SASK (ADHD Knowledge) | 0.94 | 0.96 | | 0.43 |
| Dyslexia Knowledge | 3.15 | 3.02 | | -0.66 |

*Note.* All 19 campers who completed pre-camp questionnaires are included. SDS = Social Distance Scale (measure of stigma).

**Supplemental Table 2**

*Changes in Questionnaire Scores From Pre- to Post-Camp: Comparison Between Neurodivergent & Neurotypical Campers*

|  | Neurodivergent Campers (n = 9) | | | Neurotypical Campers (n = 5) | | | Difference Between ND & NT Campers |
| --- | --- | --- | --- | --- | --- | --- | --- |
|  | Pre-Camp Mean | Post-Camp Mean | *t* | Pre-Camp Mean | Post-Camp Mean | *t* | *t* |
| Autism SDS (Stigma) | -1.54 | -1.76 | -3.46* | -1.90 | -2.00 | -2.24 | 1.24 |
| ADHD SDS  (Stigma) | -1.61 | -1.78 | -0.87 | -1.88 | -2.00 | -1.50 | 0.17 |
| Dyslexia SDS  (Stigma) | -1.73 | -1.83 | -1.73 | -1.92 | -2.00 | -1.00 | 0.21 |
| PAK-M (Autism Knowledge) | 1.14 | 1.31 | 2.00 | 1.21 | 1.48 | 2.61 | 0.73 |
| SASK (ADHD Knowledge) | 0.67 | 0.78 | 1.96 | 0.74 | 0.90 | 2.01 | 0.50 |
| Dyslexia Knowledge | 3.17 | 3.08 | -1.26 | 3.18 | 3.22 | 1.63 | 1.33 |

*Note*. *Significant at the *p*<.01 level. Only the 14 campers who completed both pe- and post-camp questionnaires are included. SDS = Social Distance Scale (measure of stigma).
